# Brazos Valley Medical Association

**Published:** 1896-10

**Authors:** 


### Society Notes.

### Brazos Valley Medical Association.

The second semi-annual meeting of the Brazos Valley Medical Association will convene at Bryan, Texas, November 10 and 11, 1896.

H. W. CUMMINGS, President,

Hearne, Texas.

E. BRITTAIN, Secretary,

Bremond, Texas.

---

DEAR DOCTOR:—It is our pleasure to extend to you a cordial invitation to the second semi-annual meeting of the Brazos Valley Medical Association, which convenes in Bryan the second Tuesday of November. The one object of our Association is the promotion and development of the healing art, the self-respect and welfare of its votaries, and the relief and benefit of human ills. We earnestly solicit your aid and co-operation in placing the Association in a sphere where its influence will yield much towards the uplifting of the standard of true medicine in the State of Texas. Only in united effort can this end be reached, for "Like clocks, one wheel another must drive, affairs by diligent labor only thrive."

Fraternally yours,
H. W. CUMMINGS, President.

PROGRAM.—TUESDAY, NOV. 10.

1. Migraine—Dr. W. H. Oliver, Bryan. Discussion, W. W. McDonald, Easterly; W. W. Pugh, Hearne.
2. Epithelioma—Dr. Daniel Parker, Calvert. Discussion, W. B. Briggs, Easterly; F. R. Collard, Wheelock.

3. Bright's Disease—Dr. J. M. Nicks, Stone City. Discussion, Geo. R. Tabor, Bryan; I. H. Brewton, Franklin.

4. Post-Partum Hemorrhage—Dr. T. G. Curry, Bremond. Discussion, W. R. Vaughan, Nesbitt; F. M. Hall, Bryan.

5. Infant Feeding and Some of its Results—Dr. G. M. Abney, Franklin. Discussion, G. H. Richardson, Hayes; W. A. Smith, Hearne.

6. Appendicitis—Dr. F. B. Collard, Wheelock. Discussion, R. S. Carrol, Calvert; L. M. Bassett, Hearne.

PROGRAM.—WEDNESDAY, NOV. 11.

7. Typho-Malarial Fever—Dr. I. P. Sessions, Rockdale. Discussion, E. Brittain, Bremond; A. Kobro, Rockdale.

8. Bone Surgery, Report of Cases—Dr. W. W. Greer, Cameron. Discussion, D. H. Bailey, Branchville; T. A. Pope, Cameron.

9. Disorders Incident to Pregnancy—Dr. J. W. Hudson, Milano. Discussion, B. F. Watkins, Bryan; O. F. Lewis, Welborn.

10. Report of a Case of Hereditary Syphilis—Dr. D. L. Peoples, Navasota. Discussion, J. A. T. Page, Wheelock; W. C. Taylor, Branchville.

11. Pneumonia—Dr. R. W. Wallis, Rockdale. Discussion, C. T. Doremus, Hearne; W. F. Sharpe, Davilla.

12. The Doctor, his Powers and Duty—Dr. T. A. Pope, Cameron. Discussion, W. H. Kirksey, Hearne; I. P. Sessions, Rockdale.

Voluntary papers and report of cases.

